# Medical and Physical Intelligence

**Published:** 1803-11-01

**Authors:** 


### MEDICAL AND PHYSICAL INTELLIGENCE.

[ FOREIGN AND DOMESTIC. ]

### Vacciolous Inoculation.

Some reports having gone abroad that the Cow-pox has been found to secure the constitution from the plague in Constantinople, and some other parts of European Turkey, Dr. Jenner begs leave to lay before the public, the evidence which he has received of this important fact. He does not deem the point clearly ascertained, but cannot forbear thinking, that every reader will see a considerable presumption in its favour in the following extract of a letter:

*From* Dr. De Carro, *of Vienna*, *to* Dr. Jenner.

"Your discovery has already produced some consequences which you surely were very far from foreseeing when you made it known to the world. I believe that I once mentioned to you, that a French physician, M. La Font, thought he had observed, that vaccinated people were not attacked by the plague. He described to me the facts which raised the suspicion: they were few, and not very conclusive, but he spoke of his new observations with modesty and prudence, and thought only that the subject deserved his further attention. Another physician at Constantinople, M. Auban, who never had any communication with M. La Font, who is of Salonica, wrote to me about a year ago, that he also had some suspicion of the cow-pox being a preservative against the plague—did not mention facts, but said, that several people had observed the same, and many vaccinated themselves as a security against the plague. Guess what was my astonishment, when a few days ago I received, through the French Ambassador at Vienna, a packet of Dr. Auban, who begins his letter with these words: — "What I had the honour of mentioning to you long ago, concerning the cow-pox being a security against the plague, as a probability, I can now, after many experiments, speak of almost as a certainty. He describes the facts summarily in his letter, and adds two *process verbaux*, signed by him and several witnesses, desiring Citizen Champagny and myself to give them every possible publicity. The proofs are,

1st. Of

1st. Of 6000 vaccinated at Constantinople, not one has taken the plague.

2d. That infants previously vaccinated have sucked, without injury, the milk of nurses infected with the plague.

3d. That an Italian physician, Dr. Valli, who went to Constantinople to study the plague, was so persuaded of the truth of the new discovery, that upon the sole security of having been vaccinated, he shut himself up in a lazaretto, and had with people attacked with carbuncles and buboes, various modes of contact, without any effect.

4th. That the same Dr. Valli inserted into his own hand, a mixture of variolous and pestilential virus, and having felt no effect from that trial, he meant the following week to insert pestilential virus alone.

5th. That Dr. Auban, having been informed that in some villages near Constantinople, the cows were subject to some eruptions on their udders; he, with several gentlemen of the French embassy, went to those villages, and found the cow-pox then existing. The report of the inhabitants was, that they had never seen the plague, or the small-pox, among them; though both these diseases made dreadful ravages in the vicinity.

Such, my dear Sir, continues Dr. De Carro, are the extraordinary facts which have been communicated to me. I have now and then corresponded with M. Lafont and M. Auban; their correspondence announces much medical information. The second, acquainting the world with such an important discovery, runs certainly a great risk, if he deceives it by false and hasty observations.

---

### *Employment of Pot-Ash with Animal Flesh, prepared and cooked for Food, instead of Muriate of Soda.*

Many instructive facts are furnished by the history of rude nations. The Indian tribes of North America furnish some very important particulars concerning the antiseptic and wholesome qualities of alkalies. Within the territory of the United States of America, many of the tribes, and more especially the parties on expeditions for war and hunting, are scantily supplied with common salt. Frequently their stock of that article is totally expended. The Delawares, Iroquois, Wyandots, Cherokees, Chickasaws, and Creeks, then season their meats with a form of pot-ash. As these people live chiefly upon animal flesh, it is a very natural inquiry by what means they preserve so perishable a material from corruption, and with what condiment they prepare it so as to render it agreeable to the palate and stomach. It is not correct, as stated by some writers, that because they preserve and eat their meat without sea-salt, that they employ no other kind of salt. The form of pot-ash the savages sprinkle on and mingle with the flesh of beasts intended to be eaten, is CLEAN WOOD-ASHES. When they mean to dry their meat for future use, they cut it into thin slices, and expose it to a pretty hot fire; and, during the operation, they either baste it with a mixture of the gravy or liquor that drops from it *and ashes*, or they powder it, from time to time, with the cleanest *ashes they can collect*. Thus they alkalize their dried bear, deer, and bison. They find that hickory and shrubby-oak (black-jack) yield the strongest and most alkaline ashes.

If they roast their meat, they are careful to dress it frequently, as it cooks, *with ashes taken fresh from the consumed wood*. This they dispose externally on every part. A portion of it dissolves, and penetrates deep among the fibres and membranes; while the rest, united with some of the animal juices, forms a sort of saline incrustation on the outside. This alkaline condiment renders the flesh remarkably more palatable; and, what is of greater importance, reconciles it to the stomach, and prevents the ill effects it would otherwise work upon the bowels. Very commonly the Indian collects fine white ashes from the fire brands into the palm of his left hand, and dips the morsels of meat into them from his right. For the same reason, the Indians, when they broil flesh, choose to put it *upon the hot coals, and oftentimes they put another layer of live coals upon the top of the slice. Thus they obtain a salting or seasoning with alkalies*. It is a common practice for an Indian to apply a bit of meat, just before he puts it into his mouth, to the surface of a brand, that it may attract the ashes adhering to it, as we dip into salt. These practices have been, and are now, commonly imitated by white men (hunters, soldiers, &c.) who remain long enough in the woods to expend their sea-salt and provisions cured therewith. The *alkali of ashes* is then resorted to, and preserves their food and their health.

---

*An Idea of the Rate at which distilled Spirits are consumed in the United States of America; from the Medical Repository of New York.*

As a medical, commercial, and political subject of inquiry, the consumption of ardent spirits is very interesting. Economists, statesmen and moralists have long deplored the abuses of these products of the still. But it is probable the cause of their regret and lamentation will not be soon removed. As long as one class of people find a profit in distilling ardent spirits from the raw materials; a second in purchasing them by wholesale, or shipping them from country to country; and a third in dealing them by retail to those who consume them; there will be distillers, merchants, and retail dealers in abundance, to satisfy the wants of him who wishes to regale himself with brandy, gin, whiskey, &c. When we consider the great amount of these articles brought to market, and reflect that they are all destined to pass into the human stomach, it becomes interesting to know minutely the rate and manner of their consumption. The following may serve as an example of what is frequently done by a labouring man in an American town, who passes for a sober citizen. As he walks out in the the morning he takes what is called a SMALL glass (half a gill) of bitters, gin, or something of the sort, at the first grog-shop he passes; and commonly takes a second whet (another half gill) before he gets to work. Generally he takes two more of these SMALL glasses of raw and clear stuff (amounting to another gill) as he returns home to get his breakfast. Thus a half pint is disposed of before eight o'clock A. M.

On going out from breakfast he takes a LARGE glass, or a whole gill; and in the middle of the forenoon another. To this is added a third as he comes home to eat his dinner. This amounts to 3 gills between eight and twelve o'clock.

He drinks another LARGE glass as he goes out to work, at or before two P. M. another in the middle of the afternoon, and a third on returning from work at six or after; making three gills more by the time his day's work is ended.

The daily quantity of distilled spirits consumed by one of these persons is as follows,

| | |
|---|---|
| Before breakfast — — — | 2 gills |
| Before dinner — — — | 3 |
| By the time the day's work is done | 3 |
| Total, — | 8 gills, |

or one quart of distilled spirits, consumed by a single labouring man in a day, besides what he drinks in porter-houses, clubs and other meetings in the evening; and the greater part of them can still keep about, and do their work, without being actually drunk. By degrees, however, it overcomes them, and they yield to the repeated and excessive stimulus of their strong draughts.

The retail cost of these doses of spirits is a heavy tax upon the individual, and amounts to as much as would buy his family bread, butter, and sweetening.

| | | |
|---|---|---|
| Morning drams, four halfgills, at 3d. | | 1s. |
| Grog before noon, three gills, at 6d. | | 1 6d. |
| Grog after noon, | ditto | 1 6 |
| | | 4 shillings, |

or half a dollar a day, spent out of his wages for rum, to be swallowed to his own ruin, and to the pinching and impoverishment of his family. There are hundreds of men who go on at this rate of drinking as long as health and money hold out. In the city of New-York there is a great number of houses where strong liquors are thus retailed. Alkohol seems to be a greater curse to Christians than opium to the Turks.

---

Dr. VAN DEN BOSCH recommends the radix lopez as a very excellent remedy in colliquative diarrhœas; he administers it, forma pulveris, in a dose of half a drachm, to be taken three or four times per day.

---

Bill

*Bill of Mortality for Portsmouth, in North America, for* 1802. *By* LYMAN SPALDING, *M. B. &c. extracted from the Medical Repository.*

| COMPLAINT. | AGE. | NO. |
|---|---|---|
| Aptha. | 4, 4 weeks. | 2 |
| Apoplexy. | 66, 33, 55, 43, 63 years. | 5 |
| Atrophy. | 55, 69, 40, 55 years. 3 m. 60 years. | 6 |
| Cancer. | 55, 63, 60 years. | 3 |
| Canker-rash. | 8, 2, 5, 7 months. 2, 16, 23, 4 years. | 8 |
| Cholera of Infants. | 6 to 24 months. | 13 |
| Cholic, Bilious | 42 years. | 1 |
| Consumption. | 14, 74, 53, 47, 53, 30, 69, 17, 33, 30, 60, 14, 33, 18, 69, 64, 60, 33, 28, 48, 52, 18, 29, 50, 63, 28, 22, 30 y. | 28 |
| Debauchery. | 55, 38 years. | 2 |
| Dropsy. | 69, 50, 84, 52, 89, 24 years. | 6 |
| Dropsy in the Brain. | 3, 7, 7, 8, 13 years. | 5 |
| Dysentery. | 3, 2, 2 years. | 3 |
| Epilepsy. | 64, 2, 2 years. | 3 |
| Fever and Ague. | 33 years. | 1 |
| Fever, Bilious. | 74, 30, 27 years. | 3 |
| Fever, Bilious Malig. | 44, 31, 41, 13, 35, 21, 30, 40, 30, 13 y. | 10 |
| Fever, Pulmonic. | 65, 45 years. | 2 |
| Gout. | 52 years. | 1 |
| Gravel. | 41 years. | 1 |
| Hooping-Cough. | 10 weeks to 1 year. | 8 |
| Infantile Complaints. | 6 days. 4 weeks. | 2 |
| Measles. | 7, 1, 20, 4, 9 m. 2, 7, 1, 2, 1, 2 years. | 11 |
| Mortification. | 7 months. 1 year. | 2 |
| Old Age. | 94, 90, 78, 76 years. | 4 |
| Palsy. | 60, 74, 64, 50 years. | 4 |
| Phrenitis. | 30, 12 years. | 2 |
| Premature Birth. | | 6 |
| Quinsy. | 3 years. | 1 |
| Scald Head. | 1 year. | 1 |
| Small-Pox, natural. | 33 years. | 1 |
| Small-Pox, inoculated. | 1 year. | 1 |
| *Casualties.* Drowned. | 48, 60 years. | 2 |
| Fall. | 55 years. | 1 |
| Frozen. | 82 years. | 1 |
| Poison by Opium. | 4 months. | 1 |
| Suicide. | 32 years. | 1 |

Portsmouth, situated 43 deg. 5 min. N. 70 deg. 41 min. W. from London, contains about 5600 inhabitants. The town has been very unheathful; some epidemic having raged the whole year. The hooping-cough, in January and February, was very prevalent; and some sporadic cases continued till September. The measles made its appearance about the middle of March, and was very very prevalent till July; at which time a bilious malignant fever made its appearance, and continued till August, when the cholera and canker-rash commenced, and continued through the year.

---

Mr. OLLENROTH, Surgeon General to the Prussian Army, has invented an apparatus for removing extraneous bodies from the œsophagus. It consists of sixty-one beads of tin, well-polished, each of the size of a large pea. At one end they are somewhat excavated, and in the middle perforated with an opening, through which a double lute-string is drawn so as to form of them a flexible cylinder, the convex part of the upper beads being received into the concavities of those under them. The knot with which the two strings are tied together must be covered with leather, and is hid in the cavity of the uppermost bead, and to it a sponge of about eight or ten inches in diameter is fastened. Five or six loops are adapted to that flexible cylinder, each made of strong silk strings, so as to form two 8 crossing each other; they are applied at a proper distance from each other, about five beads being the space between every one of them, and they must be as long as three beads. At the end opposite to that where the sponge has been fastened, a handle is adapted to the lute-strings issuing there. This instrument, on account of its flexibility and smoothness, glides easily down the œsophagus, the sponge dilating the topical contraction of that organ, while the loops serve for seizing and bringing out the extraneous body, as needles, pins, &c.

---

Mr. R. VAZIE has invented an instrument for facilitating evaporation. It consists of a hollow cylinder, caused to revolve slowly within a boiler of correspondent form and dimensions. A common roasting-jack may be so applied as to give motion to a number of these cylinders. Equal vessels being filled with water, and exposed as nearly as possible to the same degree of heat, the cylinder was applied to one of them, and it was found that the evaporation in this vessel exceeded that of the other in the proportion of three to two nearly. The heat of the agitated vessel was constantly 170°, that of the other 212°.

---

Dr. SCHAUB has discovered a new method of obtaining Prussic acid in a state of absolute purity. The process consists in pouring upon one part of Prussian-blue, half as much sulphuric acid, diluted with an equal quantity of water, and subsequent distillation. The Prussic acid passes over in alcohol; its odour greatly resembles the water of the Lauro Cerasus. It is a deadly posion to animals.

---

The following method of obtaining tungsten is recommended by RICHTER, a German chemist:—Let equal parts of tungsten oxide (tungstic acid) and dried blood be exposed for some time to a red-heat in a crucible; pass the black powder, which is formed into another smaller crucible, and expose it again to a violent heat in a forge forge for at least an hour, and tungsten will be found in the crucible, in its metallic state.

---

The Rhus radicans of Linneus, or the toxicodendron of Tournefort, the juice of which is acrid and corrosive, and which by simple contact, commonly causes erysipelatous eruptions, has been hitherto known only by its destructive qualities, and by some properties which are useful in dying. Dr. DUFRESNOY, of Montpellier, has accidentally discovered in this plant certain valuable qualities. Having observed that a young man, who had been troubled for six months with a tetter on his wrist, was suddenly cured by handling the Rhus radicans, he determined to try the effect of it in other cases; and after several experiments, he has ascertained its efficacy in destroying ring-worms, and in healing paralysis.

---

A beautiful fossile fish was lately found in one of the quarries of Nanterre, near Paris, which is the second that has been discovered in a mass of solid stone; it belongs to the genus Coryphene, of Lacepede, and has a close affinity to the Coryphenus Chrysurus.

---

The DUKE of WURTEMBURG has lately appropriated the sum of 40,000 German florins, to establish a Medico-Chirurgical Hospital, in the University of Tubingen, as likewise a Lying-in-Hospital for Women. He has, at the same time, augmented the funds of the public Library of that University.

---

Mr. Fox will commence his Lectures on the Structure and Diseases of the Teeth, in the Theatre of Guy's Hospital, on Tuesday, November 29, at seven o'clock.

---

The following gentlemen are elected Presidents of the Physical Society at Guy's Hospital for the present session: Dr Haighton, Dr. Walshman, Dr. Curry, Mr. A. P. Cooper, Mr. Thomas Hardy, and Mr. William Saunders.

---

A History of Animal Chemistry, by W. B. JOHNSON, the friend of the late Dr. Darwin, in three volumes, octavo, with a copious Index, is nearly ready for publication.

---

A new Edition of Mr. SKRIMSHIRE's popular Chemical Essays will be published next month.

